# Increasing Maximum Penalties for Animal Welfare Offences in South Australia—Has It Caused Penal Change?

**DOI:** 10.3390/ani8120236

**Published:** 2018-12-08

**Authors:** Rochelle Morton, Michelle L. Hebart, Alexandra L. Whittaker

**Affiliations:** School of Animal and Veterinary Sciences, The University of Adelaide, Roseworthy Campus, Roseworthy 5371, Australia; rochelle.morton@student.adelaide.edu.au (R.M.); michelle.hebart@adelaide.edu.au (M.L.H.)

**Keywords:** animal welfare legislation, penalties, animal cruelty, fines, imprisonment, South Australia

## Abstract

**Simple Summary:**

Evidence suggests that the South Australian public regard the penalties handed down in court for animal abuse as too lenient. Parliament responded to this concern when amending the *Animal Welfare Act 1985* (SA), and increased the maximum penalties for animal welfare offences. However, since sentencing information is not readily accessible, it is unknown whether the increases to the maximum penalties in the legislation have caused any changes to the penalties handed down in court. This study investigated this issue by analyzing closed case files gathered from the Royal Society for the Prevention of Cruelty to Animals (SA), to determine the average prison sentence and fine given for animal welfare offences. Fines and prison sentences handed down have doubled in magnitude since Parliament increased the maximum penalties. However, it remains unknown whether these increases to the average penalties are enough to effectively punish animal abusers, and if the general public is content with this outcome.

**Abstract:**

Animal welfare legislation in South Australia underwent amendments in 2008, where all the maximum penalties for animal welfare offences were doubled. This commitment to increased penalties arguably provides evidence of the legislature’s intent with respect to penalties. Studies have speculated that the legislative intent behind the increased penalties is not being reflected in the courts. This interdisciplinary research sought to gain evidence to confirm or disprove these speculations, by quantifying the average custodial sentence and monetary fine handed down in court before and after the 2008 amendments. Furthermore, trends relating to the species of animal affected and the demographics of the offender were identified. A total of 314 RSPCA (SA) closed case files from 2006 to 2018 were converted into an electronic form. Since the amendments, the average penalties have doubled in magnitude; fines have increased from $700 to $1535, while prison sentences have increased from 37 days to 77 days. Cases of companion animal abuse were most common (75% of all cases) and the location of the offence was found to influence offending. These findings suggest that the 2008 amendments have caused the average penalties to increase. However, it is debatable whether these increases are enough to effectively punish animal abusers.

## 1. Introduction

The last few decades have seen increased public concern regarding issues of animal welfare [1]. With this growing interest in animal welfare, academics and the general public have begun questioning the efficacy of animal law in the regulation of animal welfare [1,2,3,4,5,6]. Studies have identified a number of weaknesses in animal welfare legislation: the ambiguity of the language used [5], the questionable reliability of using an inadequately resourced charitable body for enforcement [5,7], the efficiency of the penalties imposed for offences [6], and the general public’s overall disappointment in the magnitude of those penalties [1,2,3,4,8]. The overall consensus is that the general public is largely in favor of harsher penalties, often in the form of jail time. These findings form the basis of the ‘public’s concern’ in relation to the penalties given to animal welfare offenders. This has led the South Australian (‘SA’) Parliament to amend the animal welfare legislation to reflect the ‘public’s concerns’ [9]. Evidence of this can be seen from statements made during consultation on the bill proposing changes to the SA *Prevention of Cruelty to Animals Act 1985* (now the *Animal Welfare Act 1985* (SA) (‘AWA’)); the Honorable Russell Wortley stated:

“Extensive consultation took place with the general public and relevant organizations over the suggested amendments to this bill to ensure that appropriate measures for the welfare of animals were enforced through the proposed legislation. It was evident throughout this consultation period that the community clearly does not accept malicious behavior towards animals, with widespread support for improved measures for the welfare of animals. The irresponsible act of causing harm to an animal is deemed as a serious offence by this community and this government. The proposed changes to this bill reflect the public’s concerns”.

Parliament listened to the public and introduced several amendments to the AWA in 2008. The amendments awarded greater power to inspectors, introduced an aggravated offence and significantly increased all the maximum penalties for animal welfare offences [10]. This commitment to increased penalties in SA arguably provides evidence of the legislative intent behind the amendments, in that Parliament intended to “get tough” on animal welfare offenders by sending a message that animal abuse would not be tolerated [11,12]. It can be assumed that this idea to “get tough” is somewhat of a theoretical objective behind the increased penalties, and the practical objective is presumably to increase sentences for animal welfare offences, since this is the view taken by commentators in the literature [13,14,15].

Studies have speculated that legislative intent behind increased penalties are not being reflected in the decisions being handed down in the Magistrates’ Courts. Boom and Ellis [13] suggested that the New South Wales criminal justice system might be failing to give effect to the legislature’s intent with respect to penalties. Similarly, Geysen, et al. [14] speculated that the penal increases within the Queensland animal welfare legislation were not being reflected in the sentencing outcomes. However, there is a need for extensive empirical research into this issue in order to confirm these speculated concerns. This is difficult to achieve as sentencing data required for this type of research are not only hard to source, but as Markham [15] noted, due to the complexity of case sentencing, the analysis of sentencing data would be a difficult task. Quantifying such a complex process to display the absolute truth is near to impossible. There is no calculation or strict mathematical formula for handing down penalties, there is human (judicial) input, as well as defendant mitigating or aggravating factors that the court will take into account. For this reason, there is minimal research using quantitative data in this area of law. In spite of this, there is value in obtaining such data; providing the limitations are considered, the data would provide a base for further discussion, research or future legislative reform.

Focusing on the South Australian jurisdiction, this interdisciplinary research sought to address this gap, and confirm or dispute current speculation about penalties. This study used a quantitative approach using scientific methods to investigate whether penalties, in the form of custodial sentences (days) and monetary fines ($), have increased over time. The aim was to quantify the average penalty sentenced before, and after the 2008 amendments to the AWA, to assess if legislative intent behind the amendments is being achieved. Furthermore, any relationship between the species of animal affected and the penalties given to animal welfare offenders were analyzed as a measure of speciesism in animal law; to assess if offences against companion animals are treated differently to offences involving farm animal offences in court. Additionally, any demographic trends relating to the offender were documented, to identify any high-risk situations relating to the commission of animal welfare offences. It was hypothesized that there would be no significant changes in magnitude of sentences given before and after the amendments. Secondary hypotheses were that offenders would receive harsher penalties for offences involving companion animals compared to farm animals, and that trends would be observed within the age and gender of the defendant, and the offence location.

This is the first research of its kind analyzing penal shortcomings in SA, and the first updated analysis of closed animal law case files since the work of Arluke and Luke [16]. This paper does not proclaim to be an exhaustive study of animal welfare law enforcement in SA but to pinpoint trends in sentencing data and identify the matters that merit further research and discussion.

## 2. Materials and Methods

### 2.1. Ethical Statement

This research was approved by the Human Research Ethics Committee of the University of Adelaide (H-2018-103) and conducted in accordance with the provisions of the National Statement on Ethical Conduct in Human Research [17]. Data were de-identified before the analysis in the non-identifiable form; rendering personal information listed in case files inaccessible to the researchers.

### 2.2. Terminology

For the purposes of this paper, ‘amendments’ refers to the changes made to the South Australian *Prevention of Cruelty to Animals Act 1985* during the formation of the *Animal Welfare Act 1985* (SA) (AWA) in 2008. ‘Maximum penalty’ refers to the maximum custodial sentences and monetary fines outlined under section 13 of the AWA, and the good behavior bonds that are considered as an agreement under section 97 of the *Sentencing Act 2017 (SA)*, where the defendant must agree to be of good behavior for a specified amount of time. Only these three forms of penalties were of interest in this study as they are most commonly imposed for animal welfare offences in SA [18]. ‘Courts’ refers to the Magistrates’ Courts, which are the lowest level of court in SA. ‘Case’ refers to the legal process involving the prosecution, being the Royal Society for the Royal Society for the Prevention of Cruelty to Animals, South Australia (RSPCA)(SA), and the defendant, being the person who has been alleged to commit an offence under the AWA. There can be multiple defendants per case. Cases involving youths (under 18) were excluded due to potential differences in sentencing in the Youth Court. ‘Closed cases’ refers to cases that have been finalized. ‘Omission’ refers to the failure to act, and ‘commission’ refers to the *act* of perpetrating an offence. ‘Section 13’ refers to the section of the AWA that defines the ill treatment of animals and the corresponding charges. ‘Charge’ refers to the formal accusation made against the defendant; there can be multiple counts of charges per case. Charges under section 13 are broken down into two subsections, section 13(1) and section 13(2), which differ based on severity (Table 1).

Aggravated/section 13(1) offences are reserved for cases where the defendant intended to, or was reckless about causing the suffering of an animal, where all elements of the offence can be proven beyond reasonable doubt in court. Whereas, the basic/section 13(2) offence is reserved for acts of cruelty or neglect where there is either no intent behind the defendant’s actions or not enough evidence to prove the intent or recklessness in court. Section 13(1) and section 13(2) currently states the following:

13—Ill treatment of animals

(1) If—
  (a) a person ill-treats an animal; and
  (b) the ill treatment causes the death of, or serious harm to, the animal; and
  (c) the person intends to cause, or is reckless about causing, the death of, or serious harm to, the animal, the person is guilty of an offence.
    Maximum penalty: $50,000 or imprisonment for 4 years.
(2) A person who ill-treats an animal is guilty of an offence. Maximum penalty: $20,000 or imprisonment for 2 years.

### 2.3. Amended Changes

Two of the most significant amendments to the AWA were the increases to the maximum penalties and the addition of the aggravated offence (Table 2).

### 2.4. Data Collection

This research was conducted in conjunction with the RSPCA (SA), as they are tasked with the bulk of animal law enforcement in SA [9]. An electronic database was created of their prosecuted cases files under the AWA. A total of 314 paper-based case files were converted into electronic form. The cases available were dated from 2006–2018, thus a targeted approach was utilized to collect sufficient amounts of data before and after the 2008 amendments. The cases were defined as either ‘pre-amendments’ or ‘post-amendments’ based on the legislation cited on the initiating process (Complaint/Investigation and Summons) filed in the Magistrates’ courts. The pre-amendments data were collected from cases finalized in 2006–2009 cases, while the post-amendment data were collected from cases finalized in 2016–2018.

Data were broken down into three categories of information based on the study aims. In quantifying average penalties, data were collected on the type of offence (aggravated or basic), as well as the definition of ill treatment most commonly used in court (serious harm/section 13(3)(a), neglect/section 13(3)(b)(iv), poor living conditions/section 13(3)(b)(i), failure to mitigate harm/section 13(3)(b)(ii)) as per section 13(3) of the AWA. Section 13(3) is stated below:

(3) Without limiting the generality of subsection (1) or (2), a person ill-treats an animal if the person—
  (a) intentionally, unreasonably or recklessly causes the animal unnecessary harm; or
  (b) being the owner of the animal—
    (i) fails to provide it with appropriate, and adequate, food, water, living conditions (whether temporary or permanent) or exercise; or
    (ii) fails to take reasonable steps to mitigate harm suffered by the animal; or
    (iii) abandons the animal; or
    (iv) neglects the animal so as to cause it harm; or
  (c) having caused the animal harm (not being an animal of which that person is the owner), fails to take reasonable steps to mitigate the harm; or
  (f) causes the animal to be killed or injured by another animal; or
  (g) kills the animal in a manner that causes the animal unnecessary pain; or
  (h) unless the animal is unconscious, kills the animal by a method that does not cause death to occur as rapidly as possible; or
  (i) carries out a medical or surgical procedure on the animal in contravention of the regulations; or
  (j) ill-treats the animal in any other manner prescribed by the regulations for the purposes of this section.

Information on the species was used for the secondary aim of documenting any observed penal trends relating to the species of animal affected. Due to the broad range of species involved in cruelty cases, a categorical approach was undertaken. For the purposes of this paper, animals were categorized into companion, farm, exotic, and wild animals, based on the definitions in Table 3.

Information on the defendant was also recorded for the secondary aim of documenting any demographic trends. Data were gathered on their ages, gender and the location of the offence. Ages were calculated based on the date the case was finalized in court. Due to the variability of the locality data, the location of the offence was categorized into the SA council regions.

### 2.5. Data Processing

**Figure 1:**
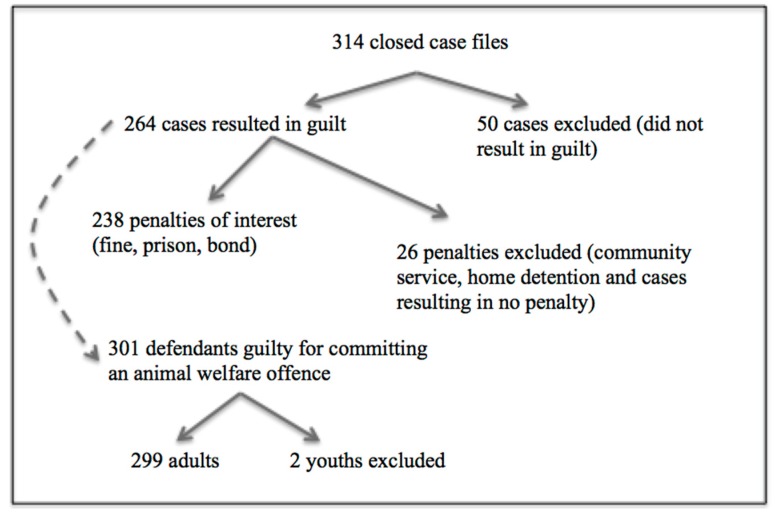
Flow chart of data processing and case exclusion.

Not all cases were deemed as ‘useable’ for this study and some had to be excluded (Figure 1). A total of 314 cases were converted into an electronic form. From these cases only 264 cases resulted in a finding of guilt (either by plea or judicial determination; 84.1%). The remaining 50 cases were withdrawn (4.8%), resulted in a caution letter (10.2%), or were found to be not guilty (0.9%). The 264 guilty cases were deemed as useable data, as a case must result in guilt, whether it is from a plea or finding, to enable the imposition of a penalty. From these guilty cases, a total of 238 penalties were sentenced with fines, imprisonment or good behavior bonds (90.1%). The remaining 26 guilty cases resulted in an alternative penalty, being community service (2.3%), home detention (0.4%) or no penalty at all (7.2%), as described in the *Sentencing Act 2017* (SA). These alternative penalties were excluded from the dataset, as they were not commonly imposed or the subject of the amended changes in 2008. Focusing only on the 264 cases that resulted in a finding of guilt, a total of 301 defendants were accused across these offences. There were more defendants than cases as some cases had co-defendants (multiple defendants per case). Two defendants were excluded from the 301 total due to the defendants being under the age of 18.

### 2.6. Data Analysis

All data were entered and analyzed in Excel 2013 (Microsoft Corp., Redmond, WA, USA). For the comparison between pre-amendment penalties and post-amendment penalties, a Chi-Squared Test was used due to the non-parametric nature of the data. Significance was determined at *p* < 0.05. Descriptive statistics were used to identify any trends within the species and demographic datasets. A heat map was created in Fusion Tables (Google LLC, Mountain View, CA, USA) to display the locality of animal welfare offences across SA.

## 3. Results

### 3.1. Changes to the Type of Penalty Imposed

**Figure 2:**
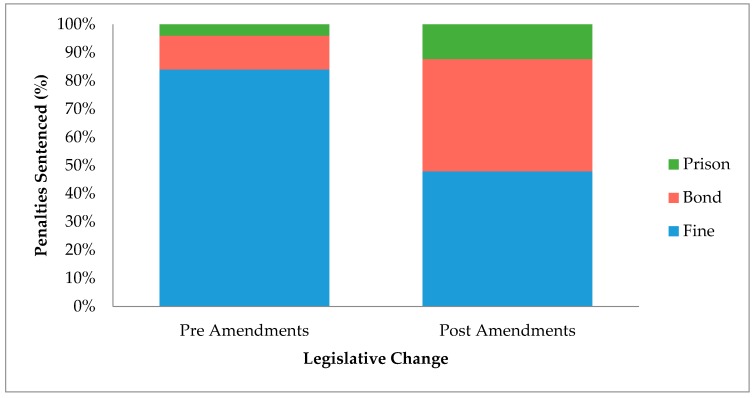
Percentage of penalties sentenced under section 13 of the AWA before and after the 2008 amendments. Pre-amendment data (*n* = 75) and post-amendment data (*n* = 163) are broken down into the three most commonly sentence penalties imposed in court: good behavior bonds, imprisonment and fines. Error bars are not included, as the data are presented as percentages; *p*-value < 0.05.

This study compared the sentencing outcomes under section 13 of the AWA before and after the increases to the maximum penalties in 2008, to assess if the intent behind the increases is being achieved through court sentencing principles. Since fines, prison sentences and good behavior bonds are most commonly used for animal welfare offences, the prevalence of these three penalties were compared before and after the amendments (Figure 2). Prior to the amendments, fines contributed to the majority of penalties, whilst good behavior bonds and prison sentences made up the minority. Since the amendments, the imposition of fines has declined while the imposition of good behavior bonds and prison sentences have both increased (*p*-value = 9.0 × 10^−7^).

**Figure 3:**
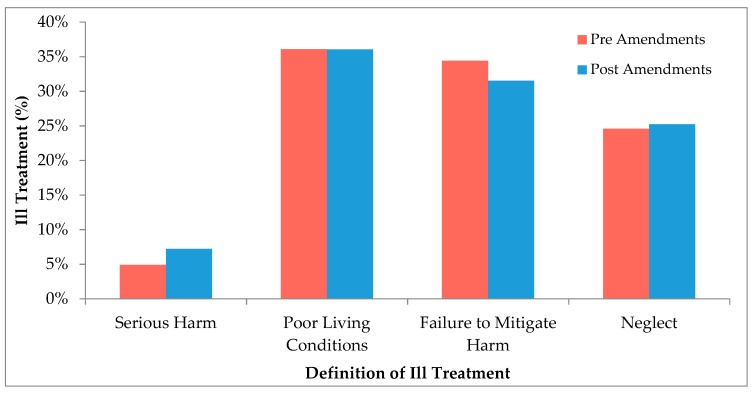
Comparison of the percentage of offences defined under section 13 of the AWA presented in courts before and after the 2008 amendments. Only the four most common definitions were considered and broken into pre-amendments (*n* = 61) and post-amendments (*n* = 222). Poor living conditions include failure to provide adequate food and water. Error bars are not included, as the data are presented as percentages; *p*-value = 0.50.

In order to further understand the decrease in number of cases resulting in fines and the increased number resulting in good behavior bonds and prison sentences, the types of ill treatment defined in the cases were compared before and after the amendments (Figure 3). All definitions of ill treatment are outlined under section 13(3) of the AWA. The most common types reported in the dataset were: serious harm; poor living conditions and failure to provide inadequate food and/or water; and failure to mitigate harm and neglect. The percentage of the categories of ill treatment defined in the cases showed no statistical association with the amendments (*p*-value = 0.50). This indicates that the type of animal welfare offences charged has remained relatively constant over the last decade.

### 3.2. Penalties Relative to the Maximum Penalty

**Figure 4:**
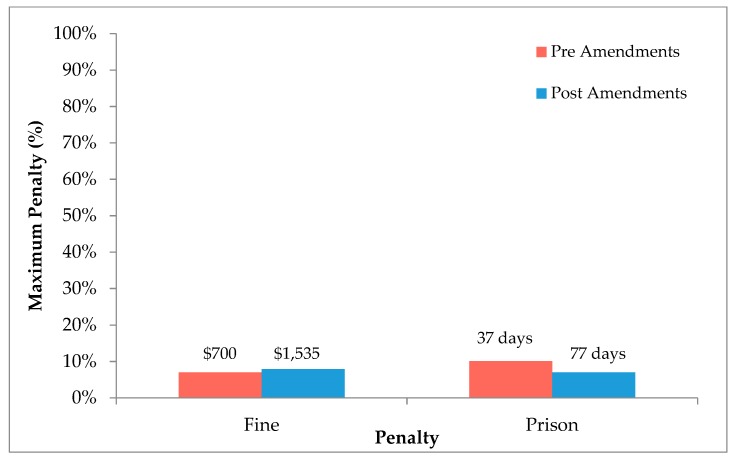
Comparison of the penalties imposed relative to the maximum penalty under section 13 of the AWA before, and after the 2008 amendments. Only prison sentences (*n* = 23) and fines (*n* = 130) were considered, as they were the subjects of the 2008 amendments. The average sentences are listed above their corresponding percentage of the maximum penalty for both pre-amendment (*n* = 62) and post-amendment (*n* = 91) data. Error bars are not included, as the data are presented as percentages.

Since the amendments, the average penalties have doubled in magnitude; fines have increased from $700 to $1535, whilst prison sentences have increased from 37 days to 77 days (Figure 4). However, when comparing penalties as a percentage of the maximum penalty no changes were observed; on average less than 10% of the maximum penalties were given both pre and post-amendment. Before the amendments, the average fine equated to 7% of the $10,000 maximum and since the amendments the average fine is now 8% of the $20,000 maximum. The average prison sentence before the amendments was 10% of the 1-year maximum sentence and has since changed to 7% of the 4-year maximum sentence.

### 3.3. Animal Species and Penalties

Average penalties for both companion animals and farm animals were compared per charge of offence (Table 4). There were a greater number of offences committed against companion animals (*n* = 167) compared to farm animals (*n* = 55), as indicated by the values in brackets. However, when comparing the average penalty given per charge of ill treatment, offenders committing offences against farm animals received a larger penalty compared to those offending against companion animals. The average fine given for offences against farm animals is $1321.13, which is almost double the $703.71 fine for offences against companion animals. While the average prison sentence for offences against farm animals is 105 days, in comparison to the average 40-day sentence given for companion animal offences. However, the 105-day sentence for farm animal offences is the average from two charges. Given this low number it is difficult to draw much in the way of a conclusion from these data. The 40-day sentence for companion animal cruelty is the cumulative effect of 35 charges. There were a total of 853 companion animals and 1685 farm animals against which offences were committed.

### 3.4. Demographic Trends

Trends in gender and age of the defendant, as well as location of the offence in our dataset, were investigated. Out of the 299 defendants involved in the 264 cases resulting in a finding of guilt, 282 defendants were charged with basic offences and 17 were charged with aggravated offences. The association between type of offence (aggravated or basic), and gender was almost significant (*p* = 0.054), where 56% of defendants committing a basic offence were males, compared to 76% for aggravated offences (Table 5).

Regardless of the severity of the offence, the average age of male defendants found guilty was 44 years old, whilst this was 39 years old for females. Taking into account the offence, the overall average age of both genders committing aggravated cruelty was 36 years old, which was younger than the average age for the basic offence, at 43 years old. Males who committed aggravated offences were on average 32 years old, which substantially differed from the female average of 51 years old.

**Figure 5:**
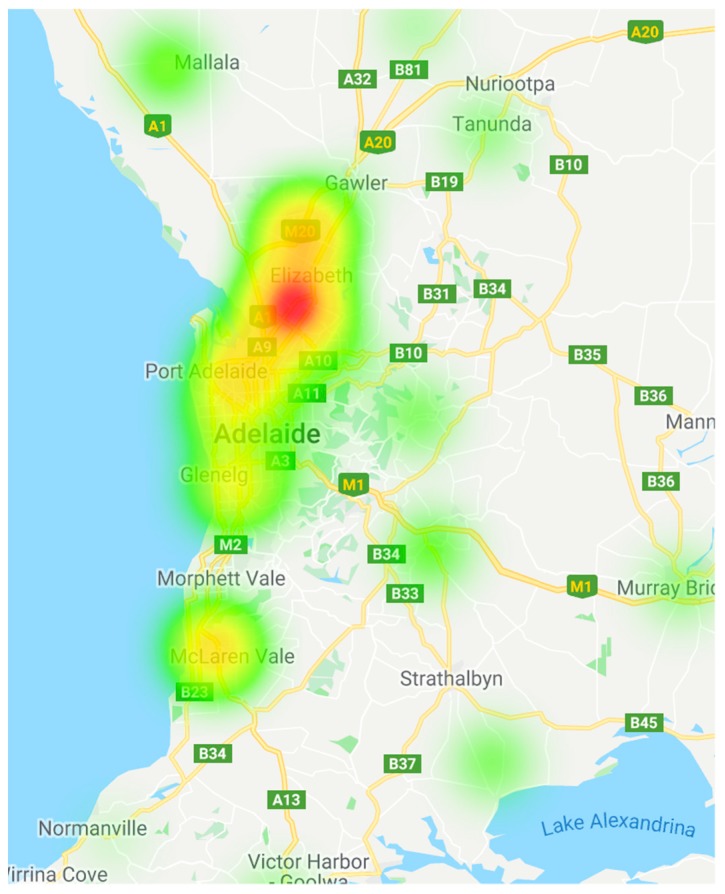
Heat map of the prevalence of animal welfare offences in South Australia’s metropolitan areas.

The location of the offence was categorized based on SA metropolitan areas (Figure 5). A much higher percentage of animal welfare offences were recorded in Adelaide’s northern suburbs (31%). The north-western suburbs had the second highest prevalence of cruelty (9%), closely followed by the southern suburbs (8%). For a more accurate comparison between the prevalence of animal welfare offences and the locality of the offence, the total numbers of animal owners in each area should be used but these data are not readily accessible.

## 4. Discussion

Since the amendments, the frequency of penalty types imposed has shifted and the average penalties have also doubled in magnitude. However, relative to the maximum penalty no changes were observed; less than 10% of the maximum penalties are being used, regardless of the amendments. This study hypothesized that the amendments to the AWA made in 2008 have caused no significant changes to the sentencing outcomes. This hypothesis can be accepted or refuted depending on interpretation. When considering the increases to the average penalties sentenced, the hypothesis would be disproven as the amendments have doubled the average sentences. However, when considering the average sentences relative to the maximum penalties, no changes were observed, which confirms the hypothesis. The remainder of this paper will examine these results in the light of available literature, including cross-jurisdictional reports.

### 4.1. Shifts in Penalties Imposed

The most common penalties handed down for animal welfare offences were fines, good behavior bonds and prison sentences. Fines are generally considered the least severe option; while imprisonment is the most severe option [13,15]. Good behavior bonds can be perceived as a middle ground between fines and imprisonment, as bonds generally have a sum and duration attached to them. Thus, for the purposes of this paper, fines are the least severe option, bonds are intermediate, and imprisonment is the most severe option. Due to the complexity of case sentencing, analyzing penal outcomes is fraught with difficulty. There is no absolute calculation or strict mathematical formula for handing down penalties; human (judicial) decision is determinative, yet defendant factors and circumstances will also be considered by the court. Judicial discretion is a valuable and important component of the criminal justice system, and this study does not seek to undermine this, or in any way suggest that this should not be the status quo. However, we do believe there is value in reporting objective data on animal law sentencing, in spite of the complexities of the sentencing process. For this reason, this study has outlined trends in the sentencing data without considering any factors that contributed to sentencing, such as early pleas, or mitigating or aggravating factors.

The types of penalties handed down in court for animal welfare offences have changed in the last decade, where the proportion of cases resulting in a fine has decreased, and the cases resulting in bonds and prison sentences have increased (Figure 2). This indicates a movement from the assumed (based on definition) less severe penalties, to the more severe penalties. This change could be attributed to two possible causes: either the offences have become more heinous since the amendments and warrant harsher penalties or, that following the amendments courts are imposing harsher penalties for offences of similar severities. In order to better understand this situation, a comparison between the prevalence of types of cruelty committed before and after the amendments was conducted (Figure 3). This analysis demonstrated that there were no differences in offence-subtype. There were more cases of negligent-type acts (omission), rather than the application of actual cruelty (commission). This finding tends to go against our first explanation for the change, and hence indicates that the courts are imposing harsher sentences. Although these results are indicative that the amendments are having an effect, we cannot be confident that these changes are due to the amendments. Changes in law, especially over a long period of time, are often multifactorial, which makes understanding the cause of change difficult. This was discussed by Freiberg and Ross [19] regarding sentencing reform in Victoria, where they stated “attempting to explain changes in the criminal justice system is an exercise fraught with peril … The causes of change are complex, and often unknowable”. This makes it almost impossible to conclude whether the changes observed in Figure 1 are due to the amendments to the AWA in 2008 or simply due to the criminal justice system changing over time.

An argument supporting the idea that courts are imposing harsher sentences is that the magistrates appeared to be aware of the penal changes, and the intent behind them. This was noted during the 2010 South Australian case of *RSPCA v Crisp* [20], where Magistrate Forrest stated:

“The usual outcome on a plea of guilty to this offence is the imposition of a fine. The fact that Parliament has set $20,000 as the maximum fine and that was the subject of an amendment within the last few years, I think, where the maximum financial penalty was raised from $10,000 to $20,000, the fact that Parliament did that reflects the concern of the community as to the ill treatment of animals. It is a matter which the community—and in this I include yourself—regard as being something that should be severely punished”.

However, this is only an example of one case out of the 165 cases sentenced after the amendments. Without examining all the case sentencing submissions, it remains unknown whether all magistrates had a similar approach to Magistrate Forrest, making it difficult to conclude that the amendments caused the penal changes. One way to understand this further would be through judicial interviews, to establish if the magistrates understood the intent behind the amendments and implemented it into their penal decisions.

However, when comparing the changes in penal trends observed in this study to the trends observed in other areas of law, similar results have been found. An analysis of New Zealand sentencing trends unrelated to animal law found that the use of fines declined, the use of imprisonment did not change and, as an alternative to imprisonment, the use of periodic detention and community service penalties increased [21]. These same trends are seen in the current study, where fines decreased, imprisonment increased, and, as an alternative penalty to imprisonment, good behavior bonds increased. These trends were also observed in the UK, where imprisonment and community-based sentences increased and fines decreased [22]. Halliday [22] interpreted these changes as a move towards tougher sentencing, which could not be ascribed to changes in the seriousness of an offence [21]. Previously fines would have been imposed for less serious offences, whereas now these offences receive a community-based order, which follows the trends observed in this study.

At the current time with the limited data available it is impossible to discern whether the shifts in penalties imposed are due to general changes within the criminal justice system, the 2008 amendments to the AWA, or a combination of both. Sentencing legislation is the subject of frequent reform [11]. For example, in SA, section 10 of the *Sentencing Act 2017*, states:

Subject to this Act or any other Act, a court must not impose a sentence of imprisonment on a defendant unless the court decides that:
  (a) the seriousness of the offence is such that the only penalty that can be justified is imprisonment; or
  (b) it is required for the purpose of protecting the safety of the community (whether as individuals or in general).

This means that in SA, imprisonment must only be used as a last resort, which contradicts the public’s belief, who are largely in favor of prison sentences for animal abuse [1,8]. The small percentage of cases that result in imprisonment (12%) are likely due to seriousness of the offence as described in section 10 of the *Sentencing Act*, and the influence of this section may also explain the increase in good behavior bonds which are given in lieu of custodial sentences.

### 4.2. Penalties Relative to the Maximum Penalty

Since the most significant amendment to the AWA was the increase to statutory maximums, it is important to assess the change in the average penalty relative to the maximum. The average length of custodial sentence, and value of monetary fines have doubled in magnitude since the amendments, however when taking into account the maximum penalties, which also doubled, there is no change observed (Figure 4). On average, less than 10% of the maximum penalties are being used in court. This has not changed over the last decade regardless of the amendments.

Although there are no empirical data available in other areas of SA law, it was suggested by a former defense lawyer and South Australian police prosecutor that this poor use of statutory maximums is not exclusive to animal law [18]. Of course, it is not realistic to expect every case of animal cruelty to be handed down the maximum penalty. However, maximums do provide the sentencing courts with a guide of the benchmark against which the gravity of that particular offence should be measured [15]. In relation to increasing statutory maximums, the change sends a message that Parliament intended to “get tough” on people who abuse animals [11,12], and it has been assumed that sterner sentences should be imposed to follow through with Parliament’s intent [13,14,15]. However, this raises a number of questions: do we really know Parliament’s intention behind increasing the statutory maximums? Was it just a symbolic gesture to signify a movement to “get tough”? Did they double the maximum penalties knowing that only 10% of the maximums would be used, but believing that it would cause the average penalties to increase? Did they even consider the practical changes that may arise from increasing the statutory maximums? All of these questions have been posed by commentators on this subject [13,14,15], but no studies have formally investigated ‘Parliament’s intent’. The likely case is that the ‘intent’ discussed in the literature is a subjective measure based on an individual’s viewpoint as to the change resulting from the amendments. In order to establish Parliamentary intent at the time of introducing the amendments, the best evidence that researchers can likely acquire would be from interviewing those Members of Parliament proposing, supporting and debating the bill as it passed through the Houses.

It could be argued that by only using a small percentage of the maximum penalty, especially after they were the subjects of amendment, sends a message to the public that animal abuse is not considered a serious social problem by the courts [23]. The evidence of the link between animal abuse and human violence suggests otherwise [24,25,26,27,28,29,30,31,32]. A growing body of literature reveals there is in fact a link between juvenile violence against animals, and later adult violence against humans. In more simplistic terms, the teenagers who actively attack the neighbor’s cat often develop into a child abuser [24,31], spouse-beater [24,25,26,27,28], or even a murderer [29,30]. The weight of this link between animal abuse and human violence should be a driver for the criminal justice system to improve sentencing outcomes in order to break this cycle at the animal stage, before it progresses further. However, arguably, increasing maximum penalties is not the most efficient way to make punishments for animal welfare offences more effective; it may be a too simplistic approach.

There is a need to establish penalties that ‘better fit the crime’ in relation to animal cruelty. A number of researchers have argued that imprisonment for criminal offences satisfies very few of the punishment theory aspects, being deterrence, incapacitation, rehabilitation, retribution, and restitution [33,34,35,36]. In the case of animal law, imprisonment only fully satisfies the retribution aspect, as the suffering experienced by the offender in jail compensates the suffering experienced by the animal harmed [34]. Other aspects of punishment seem to be ignored or are not fully met. Livingston [23] suggested that juvenile offenders should undertake psychological evaluation and treatment to reduce the likelihood of later adult violence. Sharman [6] also noted that animal abusers are acting against social norms and demonstrate moral numbness, making them a threat for future criminal activity. For this reason she recommended that more rehabilitative measures are implemented into sentencing, such as counseling and non-violent-conflict resolution training. Although in SA, Parliament had the right intentions when increasing the maximum penalties, it appears that they are not having the intended effect, whether it was to “get tough” or increase sentencing outcomes, and different approaches should be considered when punishing animal abusers. The desired outcome should not be to increase the duration or dollar value of a sentence; it should be to reduce animal cruelty through the most efficient type of penalty. Instead of wanting an animal abuser to rot away in a jail cell for a couple of years or to financially cripple them, maybe if the courts enforced mandated counseling more frequently it may actually help the offender and reduce their likelihood to reoffend. This concept of penalties that ‘better fit the crime’ has not been extensively analyzed in animal law, but is probably the most important place to start when considering the disappointment in penal outcomes.

It is important to note that punishment itself is not the only way to reduce future offending; the certainty of being caught and punished has been identified as a more effective deterrent than the severity of the punishment [37]. This study primarily focused on the severity of the punishment. This was due to limitations in ability to access data, since cruelty reports go to a national hotline, and thus require accessing a different agency’s records. Therefore, no conclusions can be drawn on any relationships between investigations versus actual charges, and incidence of animal cruelty. However, this topic does warrant further research, to understand if more investigations of animal cruelty/neglect are being conducted, and whether this translates to more charges being initiated. This is especially important after a legal reform that symbolizes the movement to “get tough”, such as occurred with the 2008 amendments to the AWA.

Just as Freiberg and Ross [19] concluded almost two decades ago, it appears that penal reforms and sentencing outcomes are loosely connected. Although, the 2008 amendments to the AWA have increased the average custodial sentences and monetary fines imposed in court, it is debatable whether this is enough to reduce animal welfare offending and whether this is the effect Parliament intended, in order to “get tough” on animal abusers. Given the evidence of the link between animal and human abuse, and the ability to be more creative in sentencing under the auspices of the sentencing legislation, perhaps different approaches to penalties are now required, with a renewed focus on rehabilitation of the offender.

### 4.3. Is Animal Law Speciesist?

Speciesism is defined as “the unjustified disadvantageous consideration or treatment of certain individuals because they are not members of a given species” [38]. In relation to this study, speciesism can be viewed as giving harsher penalties, and thus ascribing greater intrinsic value, to one species over the other. For example, it would be speciesist if offenders against companion animals received harsher penalties compared to those committing offences against farm animals, for a similar type of cruelty. This concept was explored in this study, by contrasting the average penalties received by both companion and farm animal offenders. It was anticipated that penalties given for offences against companion animals would be harsher compared to offences against farm animals. However, the opposite was observed, in that average penalties were found to be higher for offences against farm animals (Table 4).

Studies have found that the public perceives companion animals as superior to farm animals [3,4,39]. This is likely due to humans having a greater emotive response to companion animals, often viewing them as having greater intrinsic value (moral value), and farm animals having greater extrinsic value (worth to humans) [39]. As a result, it would be expected that offending against companion animals would be subject to harsher penalties. However, the contrary was observed. This may be explained by the Judge holding farm animal abusers to a higher degree due to the utilitarian nature of the farming industry [39,40]. To further explain, farmers have an ethical responsibility to treat their animals humanely, as it is their employment. In legal terms, the court will apply an objective test which considers whether the ‘ordinary, reasonable person in the defendant’s circumstances’ would have acted similarly [41]. For a professional, such as a farmer, with an expert skill in this area, the test becomes elevated to the standards of ‘the reasonable professional’ in those circumstances [42].

However, one problem with the preceding analysis is that there were not enough charges involving farm animals in the dataset to make a comprehensive comparison between the average penalties given between the two groups. The total number of farm animal charges (*n* = 55) was significantly lower than companion animal charges (*n* = 167). Also when considering this on a per animal basis, more farm animals (*n* = 1685) were the subject of cruelty charges compared to companion animals (*n* = 853). Despite the increased number of farm animals affected, less charges relating to them were being commenced via the initiating process. This is due, to the prosecution including multiple farm animals under a single charge of cruelty. This is likely due to the difficult and labor-intensive task of amassing the legal evidence needed to support the charge for each individual animal in a farm environment. Whereas in companion animal cases, generally a small number of animals are affected per case, and charges are brought on a per animal basis. Evidential burden is less since there are fewer animals, and each animal can be identified as an individual, which assists in maintaining the chain of custody. However, in reality this is a resourcing issue rather than a speciesism issue. Taking into account the paucity of data, our findings are not indicative of a speciesist element in animal law sentencing. However, in order to make a confident conclusion more data are required.

### 4.4. Demographic Trends

Establishing demographic trends in relation to animal cruelty offences will assist in resource allocation, and allow specific interventions to target groups or areas considered high-risk. Current research on demographic trends in this area either relates to the people who commit animal cruelty offences [25,43,44,45,46,47,48], or people who have a greater empathic nature towards animals [49,50,51,52,53]. These studies rely heavily on surveys and interviews with either members of the public [43,44,49,50,51,52,53], or people who were incarcerated for reasons other than animal abuse [25,45,46,47].

Currently it is accepted that males are more commonly involved in animal abuse [25,48,54,55], and that females are more empathic towards animals [48,50,52,53]. Interestingly, the findings in this study contradict these conclusions, as the proportion of males and females charged with animal welfare offences were equivalent (Table 5). However, when considering specific offences, males were charged with aggravated offences more often than females, and were notably younger. We are unable to conclude whether this reflects the actual demographics of offenders in the community, is as a result of reporting/detection differences based on the type of offences, or relates to biases in charging by prosecutors. However, at face value, this suggests that males may rely on aggression more than females, as discussed by Febres, et al. [25] when investigating animal abuse propensity by perpetrators of domestic violence. A more psychological-based research approach would be required to comprehensively understand the motives of animal abusers, and to further explain the observed gender and age effects.

A clear relationship between animal cruelty and the location of the offence was established (Figure 5). Animal cruelty was found to be more prevalent in Adelaide’s northern suburbs. This peak in cruelty may be related to the socioeconomics of the area, as Adelaide’s northern suburbs were ranked as the most disadvantaged area in SA in the 2016 Australian Census [56]. However, there is a need to establish this relationship further using more updated statistics, as well as documenting the socioeconomic status of each defendant. Interestingly though, the RSPCA (SA) does not target their education programs in the northern suburbs due to a lack of volunteers in that area [57]. It is recommended that a more targeted approach towards youth education in the northern suburbs should be established, to disrupt the high rate of offending in this area.

## 5. Conclusions

In summary, the 2008 amendments to the South Australian *Animal Welfare Act 1985* have doubled the magnitude of the average penalties handed down in court, through the doubling of the statutory maximums. It is questionable, however, whether this outcome was Parliament’s intention, and whether is it enough to “get tough” on animal welfare offenders. Although changes in severity of sentencing have arisen from the 2008 amendments, it is suspected that these changes do not go far enough to effectively deter and punish animal abusers, especially since the strong link between animal abuse and human abuse is now well established. Further research is required to determine the prevalence of animal cruelty, or at least that reported, and hence provide insight into whether the legislative changes have deterred potential offenders. Furthermore, focus should be given to more efficient ways of penalizing animal abusers to deter future offending, and to determine the best ways of preventing offending in the first place, for example through targeted education programs. The interaction between species and penalty also warrants further research, as does the relationship between animal cruelty and socioeconomics.

This is the first research of its kind analyzing penal shortcomings in animal law in South Australia. It has provided much needed information on current issues in animal law from objective data, and contributed to the limited empirical research on animal welfare legislation in Australia.

## Figures and Tables

**Table 1:** Differences between the two animal welfare charges under section 13 in the *Animal Welfare Act 1985* (SA) (‘AWA’).

Offence Characteristics	Section 13(1)	Section 13(2)
Severity	Higher	Lower
Offence Type *	Aggravated	Basic
Max. Monetary Fine	$50,000.00	$20,000.00
Max. Custodial Sentence	4 years	2 years

* Difference between offence types is the addition of mens rea (mental element) attached to the aggravated offence, where the mental element can be most commonly intent, recklessness or knowledge.

**Table 2:** Status of the maximum penalties for both aggravated and basic offences before and after the 2008 amendments to the AWA. Aggravated offence was introduced after the amendments.

Offence Type	Pre Amendments	Post Amendments
Aggravated	-	4 years imprisonment $50,000 fine
Basic	1 year imprisonment $10,000 fine	2 years imprisonment $20,000 fine

**Table 3:** Definitions and example of how species were categorized for the purposes of this study.

Category	Definition	Example Species
Companion	Animals that are under human control, providing companionship to human-owners	Dog, Rabbit, Horse
Farm	Animals commonly used for meat, eggs, milk, fur, or fibre production, providing either companionship or profit to human-owners	Sheep, Cattle
Wild	Animals that reside in the wild and are not under the control of a human-owner	Possum, Kangaroo
Exotic	Animals that are under human control, but do not fit the usual description of a ‘companion animal’	Snake, Bird

**Table 4:** Average penalties sentenced under section 13 of the AWA. Averages were calculated from the combination of pre and post amendments data and presented per charge of offence. Values in brackets indicate the number of charges.

Species Category	Fine	Prison
**Companion**	$703.71 (132)	40 days (35)
**Farm**	$1321.13 (53)	105 days (2)

**Table 5:** Percentage of males and females involved in commission of aggravated and basic animal welfare offences. Numbers were derived from the combination of pre and post-amendments data; *p*-value = 0.054.

Gender	Aggravated	Basic
**Male**	13 (76%)	159 (56%)
**Female**	4 (24%)	123 (44%)
**Overall**	17	282
